# Public awareness that HPV is a risk factor for cervical cancer

**DOI:** 10.1038/sj.bjc.6603927

**Published:** 2007-08-07

**Authors:** L A V Marlow, J Waller, J Wardle

**Affiliations:** 1 Department of Epidemiology and Public Health, Health Behaviour Research Centre, University College London, Grower Street, London, UK

**Keywords:** human papillomavirus, knowledge, awareness, public

## Abstract

We assessed awareness of human papillomavirus (HPV) in a population sample of British women (*n*=1620) using similar questions to those in a survey in 2002. Only 2.5% cited HPV as the cause of cervical cancer without prompting; up from 0.9% in 2002. Public education about HPV is urgently needed.

There has been dramatic progress in understanding the role of human papillomavirus (HPV) in the aetiology of cervical cancer (Bosch and de Sanjosé, 2003). Human papillomavirus testing is likely to play a part in screening for cervical abnormalities (Cuzick *et al*, 2006). Prophylactic HPV vaccination has been shown to be highly effective at preventing infection (Villa *et al*, 2005), and is likely to be introduced in the United Kingdom in the near future.

Despite the important public health implications of this work, knowledge about HPV in the general public appears to be low. Although surveys in Britain indicate that around 30% of young adults have ‘heard of’ HPV (Philips *et al*, 2003; Waller *et al*, 2003), this is probably little more than name recognition, because further questioning of women who say they are aware, found that fewer than half knew of the link with cervical cancer (Waller *et al*, 2003; Tiro *et al*, 2007). Using the open question ‘What causes cervical cancer*’*, the percentage mentioning HPV was less than 2% in surveys in the United Kingdom and Mexico (Lazcano-Ponce *et al*, 2001; Waller *et al*, 2004).

The potential for a prophylactic vaccine has attracted a great deal of press coverage, and this may have increased knowledge of HPV. However, internet and newspaper articles are most likely to be accessed by higher SES groups (Pew Internet & American Life Project, 2006), so the educational impact could be stronger in more educated groups.

The present study assessed awareness of HPV and knowledge of risk factors for cervical cancer in home-based interviews with a population-representative sample of British women. Responses were compared with results from a survey carried out in 2002 that used similar methods (Waller *et al*, 2004), making it possible to analyse changes over time.

## MATERIALS AND METHODS

Participants were women aged 16–97 years from a stratified random probability sampling frame. Data were collected as part of the NatCen (National Centre for Social Research) omnibus survey. Using the Post Office Address File (PAF), 6100 addresses in England, Wales and Scotland were selected for recruitment between November 2006 and February 2007. The selected addresses were sent a letter informing them about the research. Data were collected during face-to-face computer-assisted interviews.

Women were first asked an open question; ‘Thinking of cervical cancer, what do you think is its main cause’. This was followed by ‘What, if any, are other causes of cervical cancer’. Women were prompted using the phrase ‘anything else’ to encourage multiple responses. This was the same format as used in the 2002 survey (Waller *et al*, 2004). After that, women were asked a ‘closed’ awareness question: ‘Before this interview, were you aware of HPV’. This was asked after the open question to avoid it influencing their answers. Demographic information, including age, ethnicity, educational attainment and income, was also collected.

Data were analysed using SPSS version 14.0. To assess associations between demographic factors and HPV awareness, each variable was entered individually into a logistic regression analysis. Data from the recall question were recoded so that any mention of a risk factor was included in the analysis and results were merged with the data from 2002 to analyse changes over time. Chi-square (*χ*^2^) analyses revealed some socio-demographic differences between the two samples, so log-linear analysis was used, controlling for potentially confounding variables.

## RESULTS

There were 5585 eligible addresses at which 2981 productive interviews were carried out (response rate=53.4%). The questions in this survey were only asked in interviews with women (*n*=1620), but the recruitment method meant that response rates could only be calculated for both sexes. Respondents were aged 50 years on average and most were white (94%). See Table 1 for a breakdown of sample characteristics.

### Awareness of HPV (recognition)

On the basis of the simple question about awareness of HPV that was asked at the end of the interview (the term ‘HPV’ was not used previously by the interviewer), a quarter of participants (24.2%, *n*=388) said they were aware of HPV. There were some age differences, with 29% of respondents who were in the cervical cancer screening age (25–64 years) reporting awareness of HPV compared with only 15% aged 16–24 years or 65 and over. Differences in HPV awareness by ethnic group were not significant, but awareness was lower in respondents with lower levels of education and income (see Table 1).

### Recall of cervical cancer risk factors

Responses to the ‘open’ question about causes of cervical cancer are shown in Table 2. Around half of respondents answered ‘don’t know’ (51%), and only 2.5% mentioned HPV. Compared with those with no formal education, respondents were more likely to mention HPV if they were educated to A-level standard (OR=3.75, CI 1.05–13.41) or had a University degree (OR=10.28, CI 3.37–31.36). Respondents were also more likely to mention HPV if they had an annual income greater than £30 000 than if their income was less than £10 000 (OR=5.66, CI 2.34–13.72).

Overall, 28% of respondents identified something associated with sexual activity: multiple sexual partners (14%), unspecified sexually transmitted virus/infection (7%), sex at a young age (7%), frequency of sex (3%), not using condoms (3%) or unspecified sexual activity (4%). Other sexually transmitted diseases (wart virus, genital warts, herpes, chlamydia and HIV/AIDS) were cited by 2% of respondents. Additional responses included a virus/infection not specifying transmission type (5%), smoking (7%), and not going for regular screening (5%).

### Change in knowledge of cervical cancer risk factors over time

Respondents from 2007 had slightly higher incomes (*χ*^2^(3)=9.36, *P*=0.025) and were less likely to be in ethnic minority groups (*χ*^2^(4)=14.36, *P*=0.006) than respondents from 2002. Further analyses controlled for these variables.

Compared with results from 2002, there was a modest but significant increase in the number of respondents citing HPV as a risk factor for cervical cancer (from 0.9 in 2002 to 2.5% in 2007), and an increase in the number of respondents who mentioned virus/infection (from 2 to 5%). However, fewer respondents mentioned other factors relating to sexual activity, including number of sexual partners (2002: 30%, 2007: 14%), sex at a young age (13–7%), and not using condoms (7–3%). There were also significant decreases in citing wart virus (2–1%), genital warts (2–1%), not attending for smears (15–5%), smoking (15–7%), the pill (7–1%), older age (2–1%) and family history of cervical cancer (16–8%).

## DISCUSSION

In this survey, a quarter of respondents answered ‘yes’ to the question ‘Were you aware of HPV’; which is similar to previous findings in selected samples in the United Kingdom (Waller *et al*, 2003). As predicted, awareness was lower in those with less formal education, similar to disparities observed in knowledge about risk factors for other preventable cancers such as lung and skin (Viswanath *et al*, 2006).

Despite relatively high HPV name recognition, only 2.5% of respondents could spontaneously name HPV as a cause of cervical cancer, although this represents nearly a three-fold increase since 2002. There was also an increase in the proportion of respondents who mentioned virus/infection as a risk factor, but no increase in mention of ‘a sexually transmitted’ virus/infection. These results suggest that information about the viral aetiology of cervical cancer has trickled into the population since 2002, but understanding of how the virus is transmitted has not improved. It is striking that despite all the publicity about HPV, the percentage of the population who can recall that a viral infection is involved in cervical cancer, let alone which infection, is still extraordinarily low (<10%). What has also emerged, as predicted, is a significant SES gradient in awareness of HPV.

Public understanding of HPV is necessary to ensure informed consent for vaccination and testing. There is therefore an urgent need for educational programmes. Given that lower SES is known to be associated with lower attendance at cervical screening (e.g., Webb *et al*, 2004), raising awareness about HPV in these groups is particularly important. Health education initiatives should include specific efforts to reach lower SES groups, who have the lowest awareness but are at greatest risk.

## Figures and Tables

**Table 1:** Demographic characteristics of the sample and associations with HPV awareness (‘aware of HPV’)

	**Awareness of HPV**		**Significance**	
	**%**	**N**	**OR (95% CI)**	***P*-value**
*Age*				
16–24 (*n*=138)	15.2	21	1.00	
25–34 (*n*=260)	27.0	70	2.06 (1.20–3.54)	0.008
35–44 (*n*=307)	29.0	85	2.16 (1.28–3.67)	0.004
45–54 (*n*=262)	30.8	80	2.48 (1.45–4.22)	0.001
55–64 (*n*=252)	29.9	75	2.37 (1.39–4.06)	0.002
65–74 (*n*=210)	18.9	39	1.30 (0.73–2.33)	0.374
75+ (*n*=191)	9.6	18	0.59 (0.30–1.16)	0.128
				
*Ethnicity*				
White (*n*=1521)	24.2	366	1.00	
Asian or mixed Asian (*n*=38)	23.7	9	0.97 (0.46–2.07)	0.941
Black or mixed black (*n*=35)	17.6	6	0.67 (0.28–1.63)	0.379
Other (*n*=15)	20.0	3	0.78 (0.22–2.79)	0.783
				
*Education*				
No formal qualifications (*n*=536)	12.7	67	1.00	
CSE/O-level (or equivalent) (*n*=466)	17.5	81	1.46 (1.03–2.07)	0.035
A-level (or equivalent) (*n*=219)	27.1	59	2.55 (1.72–3.79)	<.001
Higher education below degree (*n*=177)	49.4	87	6.73 (4.55–9.95)	<.001
Degree (*n*=209)	44.9	93	5.61 (3.86–8.17)	<.001
				
*Respondent income*				
< £10 000 (*n*=763)	18.9	144	1.00	
£10 000-£19 999 (*n*=357)	24.8	88	1.41 (1.05–1.91)	0.025
£20 000-£29 999 (*n*=150)	42.3	63	3.14 (2.16–4.55)	0.000
>£30 000 (*n*=74)	46.6	34	3.74 (2.28–6.12)	0.000
Missing (*n*=276)	22.1	59	1.22 (0.86–1.71)	0.263

**Table 2:** Recall of risk factors for cervical cancer in 2002^a^ and 2007

	**2002 (*n*=1093)**		**2007 (*n*=1620)**		
	**%**	**N**	**%**	**N**	***χ*^2^ for difference over time (*P*-value)^b^**
*Sex-related factors*					
Wart virus	1.6	18	0.8	13	5.69 (0.017)
Genital warts	1.9	21	0.7	12	8.45 (0.004)
Human papillomavirus (HPV)	0.9	10	2.5	41	9.39 (0.002)
Herpes virus	0.9	10	0.6	10	NS
Chlamydia infection	1.5	16	0.9	14	NS
HIV/AIDS	0.7	8	0.4	6	NS
A sexually transmitted infection/disease (unspecified)	5.9	64	6.6	107	NS
A virus/infection/disease (unspecified)	2.1	23	5.4	87	21.48 (<0.001)
Having many sexual partners	29.6	324	13.8	224	99.54 (<0.001)
Becoming sexually active at a young age	12.8	140	7.4	120	24.45 (<0.001)
Having more frequent sex	4.7	51	2.9	47	6.07 (0.014)
Not using condoms	7.0	77	3.1	50	19.41 (<0.001)
Sexual activity (unspecified)	5.7	62	4.5	73	NS
					
*Lifestyle factors*					
Smoking	15.3	167	6.8	110	41.98 (<0.001)
Taking the pill	7.0	77	1.2	19	60.68 (<0.001)
Not going for regular screening (smear tests)	15.4	168	4.7	76	74.81 (<0.001)
Having many pregnancies/children	1.4	15	0.7	11	NS
Low-fibre diet	0.5	5	0.9	14	NS
High-fat diet	1.1	12	1.3	21	NS
Low-fruit and/or -vegetable diet	1.1	12	1.4	23	NS
Being overweight	0.9	10	0.7	12	NS
Stress	0.9	10	0.9	15	NS
					
*Biological factors*					
Immunosuppression	0.3	3	0.3		NS
Older age	2.1	23	1.0	17	NS
Younger age	0.6	7	0.2	3	NS
Family history (a blood relative) who has/had cervical cancer	15.5	169	8.3	134	31.82 (<0.001)
Family history (a blood relative) who has/had cancer	6.2	68	3.1	51	13.55 (<0.001)
					
*Other*					
Fate/chance/bad luck	4.3	47	2.5	41	6.49 (0.011)
Nothing	0.5	5	11.8	191	141.67 (<0.001)
Other	9.9	108	5.6	90	95.81 (<0.001)
Don’t know	32.1	350	50.9	819	1202.74 (<0.001)
Refusal	0.2	2	0.6	10	NS

NS=non-significant, *P*>0.05.

a Results reported in Waller *et al* (2004).

b Analyses controlled for income and ethnicity.
